# Primary Cutaneous Secretory Carcinoma: A Case Report and Literature Review

**DOI:** 10.7759/cureus.34203

**Published:** 2023-01-25

**Authors:** Karthik Pittala, Shannon Hall, Mallorie L Huff, Hina Sheikh, Sean J Wallace

**Affiliations:** 1 Plastic and Reconstructive Surgery, Lehigh Valley Health Network, Allentown, USA; 2 Medical School, University of South Florida Morsani College of Medicine, Tampa, USA; 3 Pathology and Laboratory Medicine, Health Network Laboratories, Allentown, USA

**Keywords:** secretory carcinoma, secretory carcinoma of the skin, cutaneous secretory carcinoma, mammary analog secretory carcinoma, secretory breast carcinoma

## Abstract

Cutaneous secretory carcinomas (CSCs) are primary neoplasms of the skin that have been just recently described in the literature through case reports and series. In this case, a cutaneous lesion was found on the left temporal region of an 83-year-old male. He was referred to plastic surgery for complete excision, with negative margins confirmed by pathology. Histology, immunostaining, and genetic testing showed characteristics confirming the diagnosis of CSC and were supported by the information present in the current literature. Our patient showed no evidence of nodal disease or recurrence during regular follow-ups. Given the rarity of CSCs, we aim to present our experience regarding the diagnosis, pathological analysis, and management of our patient as well as summarize the present literature to further open avenues of research.

## Introduction

Cutaneous secretory carcinomas (CSCs) are primary neoplasms of the skin that share similar pathological characteristics to mammary-analog secretory carcinomas (MASCs) and secretory breast carcinomas (SBCs). This novel tumor has only recently been identified in case reports and case series, first appearing in the literature in 2009 [1]. Although the axilla is the most common location for CSC, its presence throughout the body has been reported [2]. A key genetic identifier in the literature is the (12;15)(p13;q25) translocation which results in the *ETS variation transcription factor 6-neurotrophic tyrosine receptor kinase 3* (*ETV6-NTRK3*) gene fusion, a genetic property present in CSC, MASC, and SBC tumors [3].

In this report, we describe a unique CSC with microcystic morphology, positive immunostainings, such as S100, SOX10, and PanTRK, and the presence of an *NTRK3* gene recombination, similar to previous MASC and CSC reports, found on the left temporal region of an 83-year-old male without any radiographic evidence of nearby primary salivary gland tumor or nodal disease.

## Case presentation

**Figure 1:**
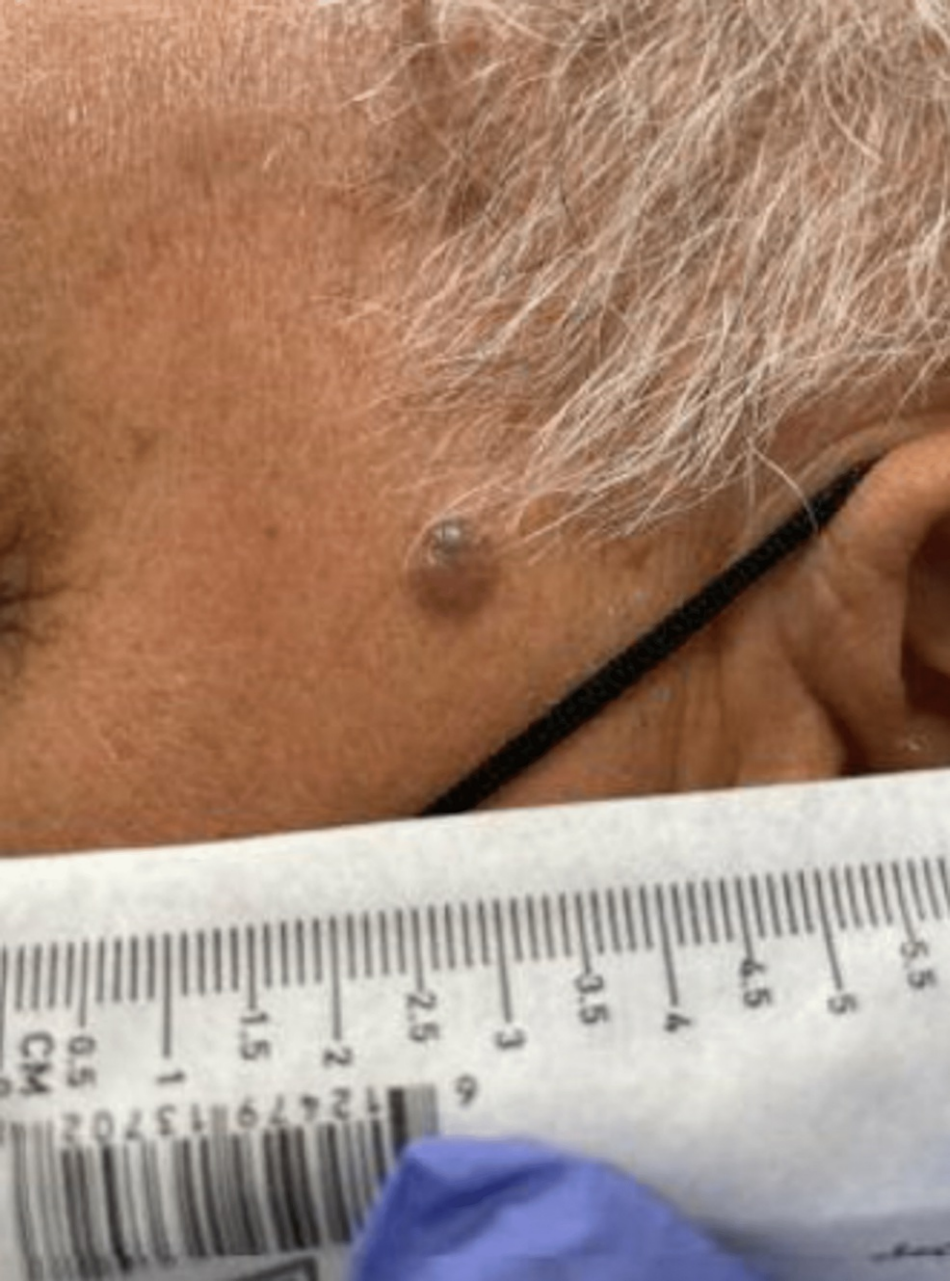
Gross appearance of the cutaneous secretory carcinoma lesion.

An 83-year-old Hispanic male with a past medical history of temporal arteritis and numerous tan, flat lentigines on his face and arms presented with a one-year history of a gradually growing, non-pruritic, and non-painful left temporal skin lesion. The lesion initially presented as a small red bump that became larger and darker over time. On presentation, the lesion was a round, raised, firm, and fixed papule measuring approximately 7 mm × 7 mm with an irregular but smooth surface (Figure 1). The papule had a reddish hue with a small area of dark blue/purple coloration. DermLite DL4W Dermoscopy (DermLite LLC, San Juan Capistrano, CA, USA) in both polarized and non-polarized modes showed mixed features, predominantly milky red with yellow globules, a somewhat rhomboid configuration, and occasional white lines. The dark blue/purple area of the lesion contained multiple adjacent blue globules.

**Figure 2:**
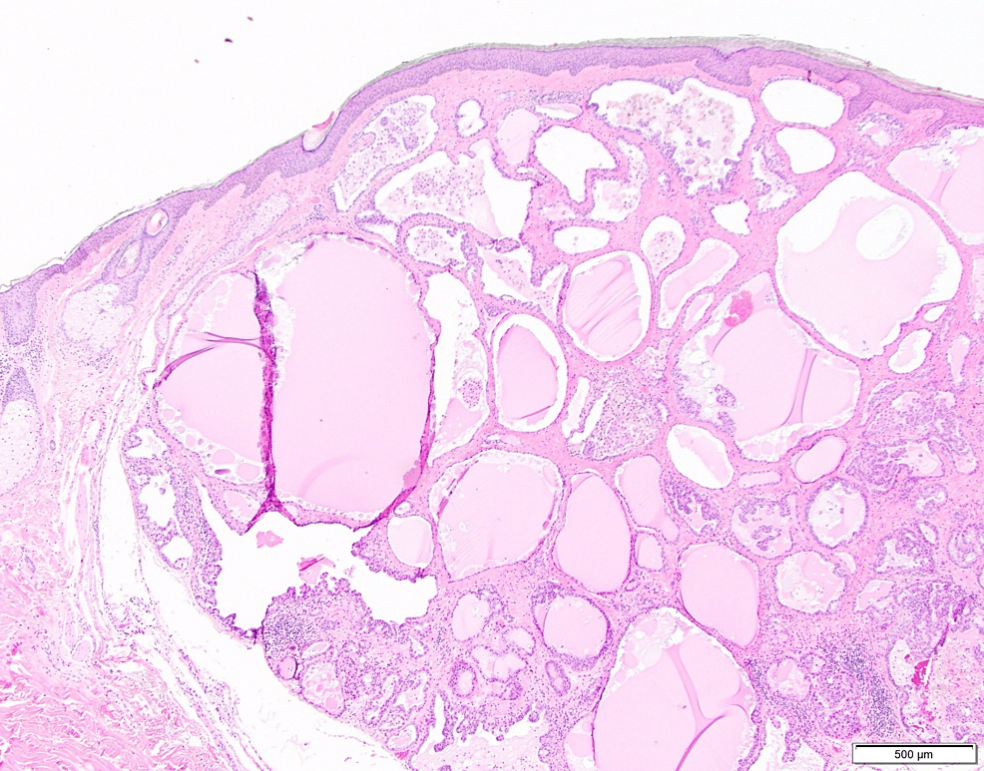
Multi-cystic circumscribed tumor in the dermis at 4× magnification with colloid-like material in the center of spaces and areas of papillary architecture.

**Figure 3:**
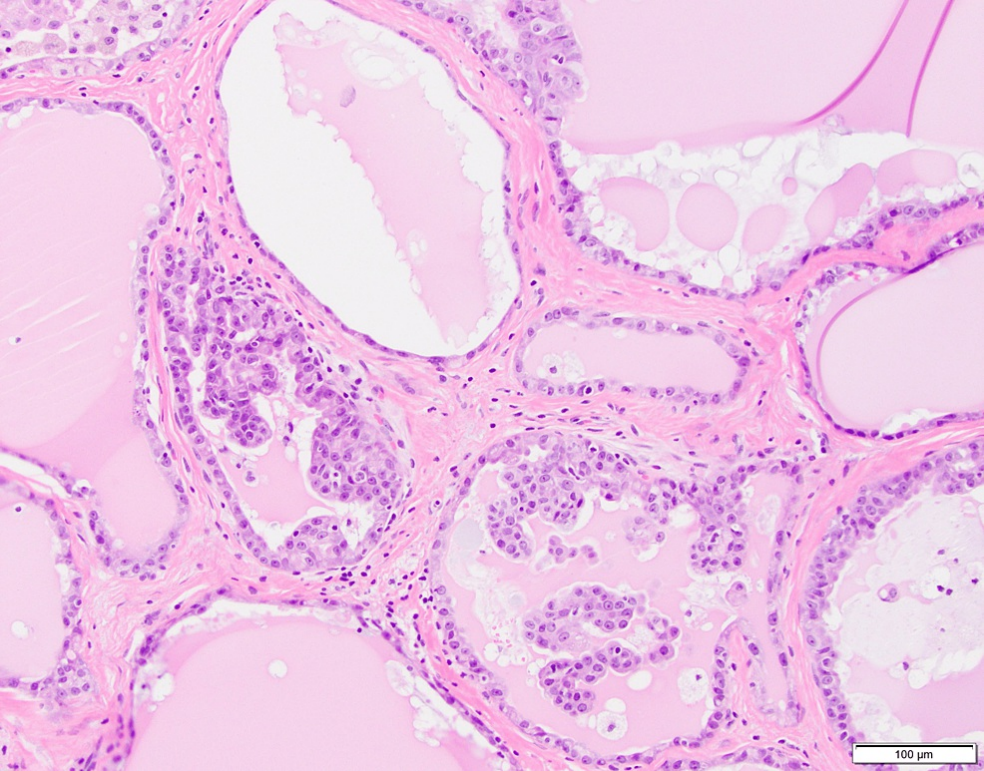
Low-grade cytology and eosinophilic cytoplasm of tumor cells at 20× magnification.

On histology, the tumor was fully circumscribed in the dermis and contained a multi-cystic and patchy micropapillary architecture (Figure 2). There was variable mono- to multi-layered lining and bland-to-mildly enlarged cytology, with central prominent eosinophilic secretions, a common characteristic of secretory carcinoma [4] (Figure 3).

**Figure 4:**
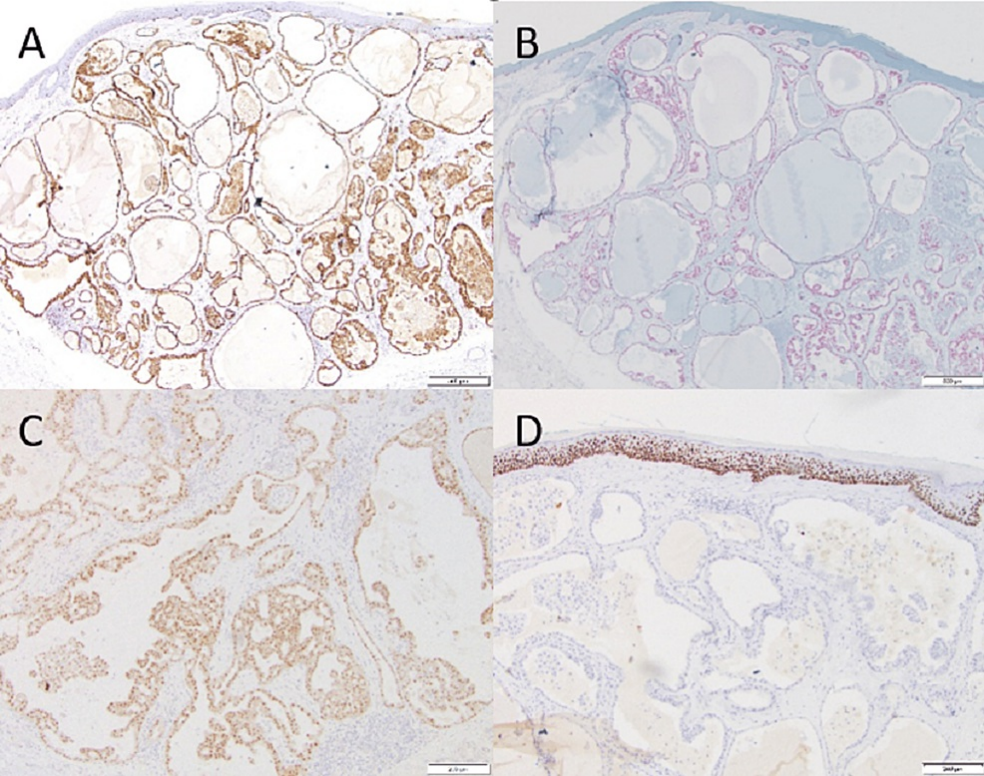
(A) Diffuse staining on S100 immunostaining at 4× magnification. (B) Strong staining on SOX10 immunostaining at 4× magnification. (C) Strong expression of PanTRK protein at 10× magnification. (D) Absence of myoepithelial cells on p40 stain at 10× magnification.

Immunostaining demonstrated strongly positive S100 staining (Figure 4A), a common characteristic of CSCs, MASCs, and SBCs [3]. Tumor cells were positive for cytokeratin 7 (CK7), epithelial membrane antigen (EMA), SOX10 (Figure 4B), gross cystic disease fluid protein (GCDFP), and estrogen receptor (ER) (about 20% of cells with variable intensity). Strong expression of PanTRK protein was also noted (Figure 4C). Myoepithelial cells were not visualized on smooth muscle actin, p40 (Figure 4D), and p63 stains. Synaptophysin, chromogranin, mucin, CK20, and thyroid transcription factor-1 (TTF-1) were negative. Break apart fluorescence signal pattern for *NTRK3* was observed in 40% of nuclei, with a normal reference range of 11.6%, and an abnormal signal pattern in 16% of nuclei, with a normal cut-off of 9%, indicating *NTRK3* rearrangement and deletion of the 5' region of *NTRK3*.

The patient was referred to a plastic and reconstructive surgeon for definitive management with complete excision. The lesion was excised with 5 mm margins and closed primarily. Negative margins were later confirmed. The patient had an uncomplicated postoperative course and was referred to surgical oncology for follow-up. No evidence of salivary tumor or nodal disease was identified on radiographic imaging. The patient is alive and disease-free after five months of follow-up.

## Discussion

This case further highlights the presence of secretory carcinomas, although typically reported in the breast and salivary gland, in the skin. Literature on this rare tumor is scarce, and the identification of key pathology is an area for future research. Through a review of the present literature on CSC, we found the mean age of patients was 50.3 years, and the median was 44 years (range: 13-98 years), with 29.2% of patients being male. The most common area of the presentation is the axilla, but other locations include the neck, groin, thigh, nipple, cheek, and eyelid [2] (Table 1). Our patient’s lesion was located on the left temporal region, a relatively rare location when compared to other reports (Table 1).

**Table 1:** Demographic information, location of tumor, tumor histology and cytology, immunohistochemical staining, ETV6-NTRK rearrangement, treatment, and clinical follow-up of reported cutaneous secretory carcinoma in the literature, including the present case. ETV6-NTRK3 =* ETS variation transcription factor 6-neurotrophic tyrosine receptor kinase 3*; ER = estrogen receptor; PR = progesterone receptor; EGFR = epidermal growth factor receptor; CEA = carcinoembryonic antigen; GCDFP-15 = gross cystic disease fluid protein 15; CK = cytokeratin; EMA = epithelial membrane antigen; TTF-1 = thyroid transcription factor 1; SLNM = sentinel lymph node mapping; RT-PCR = reverse transcription polymerase chain reaction; FISH = fluorescence in situ hybridization

Publication	Year	Age/Sex	Location	Morphology	Mitosis/Nuclear atypia	Immunohistochemistry	ETV6-NTRK3 fusion	Treatment	Follow-up
Brandt et al. [1]	2009	13/Female	Axilla	Solid and microcystic	Rare mitoses	Positive: ER, PR, S100, EGFR Negative: HEGFR2, CEA, GCDFP-15	- FISH	Excision and re-excision with SLNM	6 months
Kazakov et al. [4]	2010	40/Male	Flank	Microcystic and tubular	Mild pleomorphism	Positive: S100 Negative: Calponin, α-smooth muscle actin	- FISH	Excision	4 years
Albus et al. [5]	2015	64/Male	Anterior neck	Solid, microcystic, and ductal	Mild pleomorphism, rare mitoses	Positive: CK7, CAM5.2, EMA, CEA, MGA, MUC1, Ber-EP4, p63, vimentin Negative: S100, chromogranin, synaptophysin, GCDFP-15, calponin	- FISH (ETV6 deletion in 25% of cells)	Biopsy and excision	Unknown
Hyrcza et al. [6]	2015	40/Female	Axilla	Microcystic	Rare mitoses	Positive: MGA, MUC4, SOX-10, S100 Negative: GCDFP-15, ER, PR, Her2, DOG-1, p63, TTF-1	+ RT-PCR	Excision and re-excision	Unknown
Amin et al. [7]	2016	40/Female	Forearm	Solid, microcystic, and papillary	No mitoses	Positive: S100, CK8/18, CK7 Negative: ER, PR, her2/neu, EMA, CEA, p63, podoplanin, α-smooth muscle actin, calponin, PAX8, TTF-1	+ RT-PCR	Excision	6 months
Huang et al. [3]	2016	22/Female	Axilla	Microcystic	Mild pleomorphism, rare mitoses	Positive: CK7, CK5/6, AE1/AE3, CAM5.2, MGA, S100, vimentin, GATA-3, GCDFP-15, CD10, Muc-1, Muc-4 (sparse AR, PR, ER) Negative: p53, CEA, HER-2	+ RT-PCR	Excision	12 months
Chang et al. [8]	2016	57/Male	Axilla	Microcystic and tubular	Mild pleomorphism, no mitoses	Positive: AE1/AE3, CAM5.2, CK7 Negative: Ber-EP4, CEA	+ FISH	Excision	36 months
Bishop et al. [9]	2017	71/Female	Ventral neck	Microcystic	Range: 0-2/10 HPF Mean: 0.7	Positive: S100, MGA, STAT5	+ FISH	Excision	15 months
		56/Male	Axilla	Microcystic	Range: 0-2/10 HPF Mean: 0.7	Positive: S100, MGA, STAT5	+ FISH	Excision	Unknown
		24/Male	Axilla	Microcystic	Range: 0-2/10 HPF Mean: 0.7	Positive: S100, MGA, STAT5	+ FISH	Excision	77 months
		44/Female	Cheek	Microcystic	Range: 0-2/10 HPF Mean: 0.7	Positive: S100, MGA, STAT5	+ FISH	Excision	Unknown
		39/Female	Axilla	Microcystic	Range: 0-2/10 HPF Mean: 0.7	Positive: S100, MGA, STAT5	+ FISH	Excision	Unknown
		46/Female	Axilla	Microcystic	Range: 0-2/10 HPF Mean: 0.7	Positive: S100, MGA, STAT5	+ FISH	Excision	Unknown
Moore et al. [2]	2017	79/Male	Lip	Solid and microcystic	No mitoses	Positive: MGA, pan-cytokeratin stains, SOX-10, S100 Negative: p63, CD31, Mart-1	+ FISH	Shave biopsy and excision	4 months
Llamas-Velasco et al. [10]	2018	34/Female	Groin	Microcystic	Few mitoses, mild pleomorphism	Positive: CK7, CAM5.2, mammaglobin, S100 Negative: MYB, CD117, GATA3, CK20, podoplanin, calponin, CDX2	+ FISH	Excision	144 months
Kastnerova et al. [11]	2019	75/Female	Axilla	Microcystic and tubular	Few mitoses (4/mm^2^)	Positive: S100, STAT5, MGA, GATA3	+ FISH and RT-PCR	Excision and re-excision	12 months
		98/Female	Axilla	Microcystic and tubular	Rare mitoses (1/mm^2^)	Positive: S100, STAT5, MGA	+ FISH	Excision	2 months
		67/Female	Neck	Microcystic and tubular	No mitoses	Positive: S100, STAT5, MGA, GATA3	+ RT-PCR	Excision	32 months
		73/Male	Lower eyelid	Solid and papillary	Few mitoses (2/mm^2^)	Positive: S100, MGA, GATA3, CD117, TTF-1, STAT5	+ FISH	Excision	6 months
		57/Female	Thigh	Microcystic, tubular, and mucinous	Few mitoses (4/ mm^2^)	Positive: S100, STAT5, MGA, CK7, GATA3, p63 Negative: CD117	- RT-PCR + NFIX-PKN1	Excision	14 months
		75/Female	Nipple	Microcystic and tubular	No mitoses	Positive: S100, STAT5, MGA	+ FISH	Partial mastectomy with SLNM	Unknown
Tsutsui et al. [12]	2020	40/Female	Upper eyelid	Solid and papillary	Unknown	Positive: S100, SOX10, PAN-TRK stains	+ FISH	Excision and re-excision	3 months
Grinnell et al. [13]	2021	22/Female	Breast	Solid and microcystic	Few mitoses	Positive: MGA, GATA3, S100, PAN-TRK stains, ER, GCDFP-15 Negative: myosin, p40	+ FISH	Excision and re-excision with SLNM	Unknown
Taniguchi et al. [14]	2021	31/Female	Axilla	Glandular and papillary	Few mitoses	Positive: MGA, S100, GATA3, ER Negative: p63, PgR, HER2	+ Next-generation sequencing-based multiplex gene assay	Excision and re-excision with SLNM (1/4 positive)	4 years (metastasis to bilateral lungs)
Current case	2023	83/Male	Temporal	Microcystic	Unknown	Positive: SOX10, S100, CK7, EMA, GCDFP-15, ER, PAN-TRK stains Negative: TTF-1, α-smooth muscle actin, p40, p63	+ FISH (only NTRK3)	Excision	2 months

The reviewing pathologist described the tumor specimen in our case as 7 mm × 7 mm and completely circumscribed in the dermis with a depth of 1 cm. The tumor had a multi-cystic architecture, multi-layered lining, bland-to-mildly enlarged cytology, and central eosinophilic secretions. This histology closely resembles similar tumors described in the breast, parotid gland, and thyroid gland. Primary breast and parotid gland origin were excluded through proper clinical examinations and history. Immunohistochemistry staining was positive for CK7, EMA, S100, GCDFP, ER, and SOX10. The staining was negative for synaptophysin, chromogranin, mucin, TTF-1, and CK20. The negative TTF-1 excluded thyroid carcinoma. The staining was also negative for p40, p63, and smooth muscle actin, which are indicative of myoepithelial cells. The tumor expressed PanTRK through the detection of wild-type TRK protein and *NTRK *fusion gene. *NTRK3 *rearrangement was detected as well; however, *NTRK3 *fusion with the specific *ETV6 *partner gene has not been confirmed yet. Abnormality found on the break-apart probe together with the diffuse expression of NTRK protein via immunohistochemistry supports the role of *NTRK3 *in oncogenesis in this case as well [15]. *ETV6 *fusion with *NTRK3* is the most common fusion cited in the literature on CSCs and is present in most of these tumors (Table 1).

CSCs can present with a variety of morphological characteristics, with reports describing histology as a combination of microcystic, glandular, papillary, ductal, tubular, and solid growth patterns. This tumor type consistently reports a low mitotic rate, and few cases report mild pleomorphisms (20.8%). Necrosis, perineural invasion, and lymphovascular invasion are also typically not seen. Of the CSCs in the literature that underwent immunohistochemistry staining, S100 (83.3%), STAT5 (41.7%), SOX10 (12.5%), MGA (75%), GATA-3 (25%), ER (20.8%), and CK7 (25%) were most commonly positive (Table 1). The *ETV6-NTRK3* gene fusion is commonly reported as a key identifier for secretory carcinomas, including CSC, MASC, and SBC tumors. Overall, 83.3% of reports on CSC have identified the *ETV6-NTRK3* (Table 1).

Surgical excision is the primary treatment for CSC [12]. In patients where initial biopsy or excision showed positive margins, re-excision was performed. Management for our patient was the excision of the nodule with negative margins which was closed primarily. Wound management was followed up with plastic surgery and continued by the patient. Although not necessary for our patient due to negative margins and lack of sentinel lymph node involvement, entrectinib, a tyrosine-receptor kinase (TRK) inhibitor, can help patients with recurrent and metastatic disease and has been therapeutic in a case of MASC [16]. TRK inhibitors may be beneficial for our patient in the event of recurrence or metastasis, given the *NTRK3 *gene fusion that leads to oncogenic properties of the impacted cells.

CSCs are generally indolent [15], with only one definitive incidence of metastasis reported previously [14]. Our patient was followed up on postoperative day 15 with plastic surgery and at one month and two months post-excision with surgical oncology for further workup. The current plan is annual CT scanning with surgical oncology follow-up for monitoring. Close follow-up is recommended given the unknown nature of this tumor type, and precautionary imaging is recommended to identify a possible primary salivary gland source or spread to adjacent lymph nodes. The prognosis is favorable, with only one report of metastasis for MASC [17] and one definitive reported metastasis for CSC [14].

Currently, cases of CSCs are rare in the literature, so we aim to add to the current body of literature through our experience in the diagnosis, pathological analysis, and management of our patient. Accumulation of this research can open avenues for standardization in the identification of this tumor type, and, ultimately, allow for earlier diagnosis and personalized treatment.

A large single or multi-institutional study is necessary to confirm reported pathology markers and conduct genetic studies that identify CSCs. Given the rarity of this tumor type in the literature, the lack of long-term follow-up is a limiting factor in determining an accurate prognosis for patients. This emphasizes the importance of continuous follow-up and further research.

## Conclusions

CSCs are a novel tumor type related to MASCs and have only recently been reported in the literature through case reports and series. This case report supports the previous literature that CSC tumors have similar histological, immunostaining, and genetic profiles to secretory carcinomas of the breast and salivary glands. Our case is unique in the rare temporal presentation of the tumor. Although the prognosis of this tumor type is favorable, with minimal chance of metastasis and low-grade characteristics, it is an important contribution to the existing body of literature. This report and literature review aim to improve the profiling of CSC tumors to provide better patient care and help guide management strategies with a special focus on long-term outcomes.
